# Sleep duration and risk of stroke and coronary heart disease: a 9-year community-based prospective study of 0.5 million Chinese adults

**DOI:** 10.1186/s12883-023-03367-4

**Published:** 2023-09-14

**Authors:** Yiping Chen, Christiana Kartsonaki, Robert Clarke, Yu Guo, Huaidong Du, Canqing Yu, Ling Yang, Pei Pei, Rebecca Stevens, Sushila Burgess, Yujie Hua, Junshi Chen, Jun Lv, Liming Li, Zhengming Chen, on behalf of the China Kadoorie Biobank Collaborative Group, Junshi Chen, Zhengming Chen, Robert Clarke, Rory Collins, Yu Guo, Liming Li, Jun Lv, Richard Peto, Robin Walters, Daniel Avery, Ruth Boxall, Derrick Bennett, Yumei Chang, Yiping Chen, Zhengming Chen, Robert Clarke, Huaidong Du, Simon Gilbert, Alex Hacker, Mike Hill, Michael Holmes, Andri Iona, Christiana Kartsonaki, Rene Kerosi, Garry Lancaster, Sarah Lewington, Kuang Lin, John McDonnell, Iona Millwood, Qunhua Nie, Jayakrishnan Radhakrishnan, Paul Ryder, Sam Sansome, Dan Schmidt, Paul Sherliker, Rajani Sohoni, Becky Stevens, Iain Turnbull, Robin Walters, Jenny Wang, Lin Wang, Neil Wright, Ling Yang, Xiaoming Yang, Yu Guo, Xiao Han, Can Hou, Jun Lv, Pei Pei, Chao Liu, Canqing Yu, Qingmei Xia, Zengchang Pang, Ruqin Gao, Shanpeng Li, Shaojie Wang, Yongmei Liu, Ranran Du, Yajing Zang, Liang Cheng, Xiaocao Tian, Hua Zhang, Yaoming Zhai, Feng Ning, Xiaohui Sun, Feifei Li, Silu Lv, Junzheng Wang, Wei Hou, Mingyuan Zou, Ge Jiang, Xue Zhou, Liqiu Yang, Hui He, Bo Yu, Yanjie Li, Qinai Xu, Quan Kang, Ziyan Guo, Dan Wang, Ximin Hu, Jinyan Chen, Yan Fu, Zhenwang Fu, Xiaohuan Wang, Min Weng, Zhendong Guo, Shukuan Wu, Yilei Li, Huimei Li, Zhifang Fu, Ming Wu, Yonglin Zhou, Jinyi Zhou, Ran Tao, Jie Yang, Jian Su, Fang Liu, Jun Zhang, Yihe Hu, Yan Lu, Liangcai Ma, Aiyu Tang, Shuo Zhang, Jianrong Jin, Jingchao Liu, Zhenzhu Tang, Naying Chen, Ying Huang, Mingqiang Li, Jinhuai Meng, Rong Pan, Qilian Jiang, Jian Lan, Yun Liu, Liuping Wei, Liyuan Zhou, Ningyu Chen Ping Wang, Fanwen Meng, Yulu Qin, Sisi Wang, Xianping Wu, Ningmei Zhang, Xiaofang Chen, Weiwei Zhou, Guojin Luo, Jianguo Li, Xiaofang Chen, Xunfu Zhong, Jiaqiu Liu, Qiang Sun, Pengfei Ge, Xiaolan Ren, Caixia Dong, Hui Zhang, Enke Mao, Xiaoping Wang, Tao Wang, Xi zhang, Ding Zhang, Gang Zhou, Shixian Feng, Liang Chang, Lei Fan, Yulian Gao, Tianyou He, Huarong Sun, Pan He, Chen Hu, Xukui Zhang, Huifang Wu, Min Yu, Ruying Hu, Hao Wang, Yijian Qian, Chunmei Wang, Kaixu Xie, Lingli Chen, Yidan Zhang, Dongxia Pan, Qijun Gu, Yuelong Huang, Biyun Chen, Li Yin, Huilin Liu, Zhongxi Fu, Qiaohua Xu, Xin Xu, Hao Zhang, Huajun Long, Xianzhi Li, Libo Zhang, Zhe Qiu

**Affiliations:** 1 Medical Research Council Population Health Research Unit (MRC PHRU), University of Oxford, Big Data Institute, Old Road Campus, Oxford, OX3 7LF UK; 2 Nuffield Department of Population Health, Clinical Trial Service Unit & Epidemiological Studies Unit (CTSU), University of Oxford, Oxford, UK; 3 National Clinical Research Center of Cardiovascular Diseases, Fuwai Hospital, Chinese Academy of Medical Sciences, Beijing, China; 4 Department of Epidemiology and Biostatistics, School of Public Health, Peking University Health Science Center, Beijing, China; 5 Peking University Center for Public Health and Epidemic Preparedness & Response, Beijing, China; 6 NCDs Prevention and Control Department, Suzhou CDC, China; 7 China National Center for Food Safety Risk Assessment, Chaoyang District, Beijing, 100021 China

**Keywords:** Sleep duration, Prospective studies, Stroke, Myocardial infarction

## Abstract

**Background:**

There is uncertainty about the optimum sleep duration for risk of different subtypes of stroke and ischaemic heart disease.

**Methods:**

The present analyses involved 409,156 adults in the China Kadoorie Biobank study without a prior history of coronary heart disease or stroke or insomnia symptoms. The mean age of study participants was 52 years and 59% were women. Self-reported sleep duration including daytime napping was recorded using a questionnaire. The adjusted hazard ratios (HRs) for disease outcomes associated with sleep duration were estimated by Cox proportional hazards after adjustment for confounding factors.

**Results:**

The overall mean (SD) sleep duration was 7.4 (1.4) hours. The associations of sleep duration with CVD types were U-shaped, with individuals reporting 7–8 h of sleep having the lowest risks. Compared with those who typically slept 7–8 h, individuals with very short sleep duration (≤ 5 h) had adjusted HRs of 1.10 (95% CI 1.04–1.16), 1.07 (1.01–1.13), 1.19 (1.06–1.33) and 1.23 (1.10–1.37) for total stroke, ischaemic stroke (IS), Intracerebral haemorrhage (ICH) and major coronary events (MCE), respectively. Likewise, individuals with very long sleep duration (≥ 10 h) had HRs of 1.12 (1.07–1.17), 1.08 (1.03–1.14), 1.23 (1.12–1.35) and 1.22 (1.10–1.34) for the same diseases, respectively, with little differences by sex and age. The patterns were similar for all-cause mortality.

**Conclusions:**

While abnormal sleep duration (≤ 6 h or ≥ 9 h) was associated with higher risks of CVD, the risks were more extreme for those reporting ≤ 5 or ≥ 10 h, respectively and such individuals should be prioritised for more intensive treatment for CVD prevention.

**Supplementary Information:**

The online version contains supplementary material available at 10.1186/s12883-023-03367-4.

## Lay summary

- Optimum sleep duration for risk of stroke types and coronary heart disease in adults without insomnia is uncertain.
- Individuals reporting 7–8 h of sleep had the lowest risks and those who reported having ≤ 5 h or ≥ 10 h of sleep had 10% and 12% higher risks of total stroke and 23% and 22% higher risks of major coronary events (MCE), respectively.
- The findings reinforce the importance of adequate sleep duration for prevention of most cardiovascular disease types and of prioritizing adults with abnormal sleep duration for more intensive treatment for prevention of stroke and coronary heart disease.

## Background

Sleep duration is an important modifiable determinant of cardiovascular disease (CVD). Previous prospective studies, and meta-analyses of such studies, have reported that both short (≤ 6 h) and long sleep duration (≥ 9 h) were associated with higher risks of major cardio-metabolic diseases, including obesity, hypertension, diabetes mellitus, ischemic heart disease (IHD) and stroke, in addition to all-cause mortality [1–5]. Uncertainties remain about the associations of varying intervals of sleep duration with CVD and specific types of CVD (including ischemic stroke [IS] and intracerebral haemorrhage [ICH], myocardial infarction [MI] and non-MI IHD), as previous prospective studies and meta-analyses of such studies, did not include a sufficient number of cases of such diseases to reliably assess associations with sleep duration, both overall and in subgroups by sex, age or socio-economic status. Moreover, many previous studies have also been constrained by residual confounding and reverse causality biases on the observed associations due to anxiety, depression and insomnia, which influence sleep duration and associated disease risks. Moreover, the available evidence from prospective studies on sleep duration and CVD risks in diverse populations is limited, with only one large prospective study of 63,257 Chinese adults reporting that both short and long sleep duration were associated with higher risks of stroke [6]. Assessment of the associations of sleep duration, including daytime napping with major CVD types in different populations and population subgroups could enhance our understanding of the mechanisms underlying these associations and guide strategies for CVD prevention.

The aims of the present study of 0.5 million Chinese adults in the China Kadoorie Biobank were to assess: (i) the shape and strength of the associations of sleep duration with major CVD types and all-cause mortality in people without insomnia; (ii) whether the associations of sleep duration with CVD differed by age, sex, region, education, and mental health status; and (iii) whether these associations differed by presence or absence of daytime napping.

## Methods

### Study population

Details of the design, baseline characteristics and methods used to collect incident disease outcomes during follow-up had been previously reported [7, 8]. Briefly, 10 (5 rural, 5 urban) diverse regions in China were selected from China’s nationally representative Disease Surveillance Points system to maximise geographic and social diversity. A total of 1 801 200 registered residents, aged 35–74 years in each study region were identified through local residential records and invited to attend a study clinic between June 2004 and July 2008. Among the 512 713 (210,205 men and 302, 508 women) participants who completed a baseline survey (12 665 were outside the age range), but all were aged 30–79 years. Overall, approximately 30% of residents (33% in rural and 27% in urban regions) participated in the study.

### Baseline survey

At local study assessment centres trained health workers collected detailed information using laptop-based questionnaire (with automated checks to minimise errors and inconsistency) covering general demographic and socioeconomic status, lifestyle factors (e.g. diet, smoking, alcohol drinking, physical activity), prior medical history, and sleeping patterns (http://www.ckbiobank.org) was administered using a laptop direct entry system. Moreover, physical measurements were undertaken, and included height, weight, waist and hip circumference, heart rate, blood pressure, and lung function. A non-fasting venous blood sample was collected (with record of duration since last meal) for storage and onsite random plasma glucose testing using the SureStep Plus system (LifeScan).

### Assessment of sleep duration and insomnia symptoms

In the baseline questionnaire each participant was asked: “How many hours do you typically sleep per day including napping?” along with multiple choice questions: (i) Do you usually take a daytime nap? (“Yes, usually”; “Yes, but only in summer”; and “No”); (ii) Do you snore during sleep? (“Yes, Frequently”; “Yes, Sometimes”; or “No/Don’t know”). Moreover, each participant was asked whether they had any insomnia-related symptoms for at least 3 days or more in a week during the last month: (i) taking > 30 min to fall asleep after going to bed or waking up in the middle of the night; (ii) waking up early and not being able to go back to sleep; (iii) needing to take medicine (including herbal or sleeping pills) at least once a week to help sleep; or (iv) having difficulty staying alert while at work, eating or meeting people during daytime. Participants who answered “Yes” to any of the above symptoms were classified as having insomnia symptoms. Details of individual questions about sleep, insomnia symptoms, daytime napping and snoring (Sect. 10 of the baseline questionnaire) are available online http://www.ckbiobank.org/site/binaries/content/assets/resources/pdf/qs_baseline-final-from10june2004.pdf).

### Follow-up for fatal and non-fatal CVD outcomes

Study participants were followed-up on a six-monthly or annual basis, for fatal and non-fatal disease outcomes by electronic linkage via a unique personal identification number, with mortality and morbidity (for stroke, IHD, cancer and diabetes) registries and with the national health insurance (HI) systems that recorded any episodes of hospitalisation. Moreover, active follow-up was undertaken by contacting participants or their relatives on an annual basis for small proportion of individuals (~ 2%) who were not registered in the HI system.

Cause-specific mortality was monitored through China’s Disease Surveillance Points system and electronic health insurance records, with annual active confirmation of vital status through local residential and administrative records. The Disease Surveillance Points system provides reliable death registration, in which almost all adult deaths were medically certified. For the few deaths (< 5%) without medical attention prior to death, standardized procedures were used to determine probable causes of death by reviewing symptoms and signs by relevant informants (usually family members). The trained Disease Surveillance Points system staff coded all diseases on the death certificates and assigned underlying causes using the International Statistical Classification of Diseases and Related Health Problems, Tenth Revision (ICD-10). For deceased participants, the data entered into the study follow-up system (including scanned images of the original death certificates) were reviewed centrally by study clinicians, who were blinded to the study information collected at baseline. By 1st January 2018 43,291 (8.8%) participants died and 5066 (1.0%) were lost to follow-up.

The main non-fatal disease outcomes were incident IHD (ICD-10:I20-I25), intracerebral hemorrhage (ICH, I61), and ischemic stroke (IS I63), and Major Coronary Events (ICD10: I21-I23 from any source; I20; I24 or I25 only for fatal outcomes) [9]. For each specific disease, only the first events were considered. ICD-10 codes were provided by national insurance claims system and reviewed centrally by study clinicians blinded to the baseline information and standardised in accordance with international guidelines and diagnostic criteria using bespoke software [9].

### Statistical methods

The present analyses were restricted to 409,156 adult participants with no history of heart disease or stroke and no insomnia symptoms (*n* = 80,441). Cox regression was used to estimate the adjusted hazard ratios (HRs) for each disease outcome associated with sleep duration (5 groups: ≤ 5, 6, 7, 8, 9, ≥ 10 h). Models were stratified by region (10 regions) and adjusted for age at baseline (continuous), sex, smoking (never regular, ex-regular, or ever regular), alcohol intake (never regular drinker, ex regular drinker, occasional or seasonal drinker, monthly drinker, reduced intake, weekly), BMI (continuous, kg/m^2^), physical activity (Metabolic Equivalent of Task [MET] hours/day, continuous), systolic blood pressure (SBP) (continuous), SBP^2^, depression symptoms (yes/no), generalised anxiety symptoms (yes/no), snoring (yes, frequently; yes, sometimes; no/don't know), daytime napping (yes, yes but only in the summer, no). HRs for categorical variables are presented with ‘floating’ standard errors [10]. The plausibility of the proportional hazards assumption was assessed using Schoenfeld residuals and chi-squared tests based on these. The proportional hazards model was plausible for sleep duration. Subgroup analyses by (i) sex, (ii) rural or urban residence, or (iii) daytime napping were also conducted. The following sensitivity analyses were done: (i) excluding individuals with any self-reported prior disease at baseline (diabetes, hypertension, rheumatic heart disease, tuberculosis, emphysema/bronchitis, asthma, cirrhosis/hepatitis, peptic ulcer, gallbladder disease, kidney disease, fracture, rheumatoid arthritis, head injury, cancer), (ii) excluding the first 3 years of follow-up, and (iii) excluding individuals with self-rated poor health. Additional analyses were conducted comparing short (≤ 6 h) and long (≥ 9 h) compared to normal (7–8 h) sleep. All analyses were conducted using R (version 3.3.1).

## Results

Among the 489,597 participants in the CKB study before excluding those withinsomnia symptoms, the mean age at baseline was 52 years, 59% were women and 57% resided in rural areas. The present analyses were restricted to 409,156 participants with no prior CVD or insomnia symptoms. The mean (SD) sleep duration was 7.4 (1.4) hours, with 23% reporting short duration (≤ 6 h/day) and 16% reporting long (≥ 9 h/day) sleep duration, respectively. Compared to those who reported sleeping on average for 7–8 h, those reporting short or long sleep duration were more likely to be women, to have lower levels of education, lower income or to live in rural areas (Table 1). Moreover, individuals with short sleep duration were more likely to report physical illnesses (e.g. non-CVD disease, diabetes or hypertension) or have mental disorders (MDE and GAD). However, there were little differences in the prevalence of smoking or alcohol consumption by sleep duration, nor in the mean levels of BMI, SBP, physical activity and lung function (Table 1).

**Table 1:**
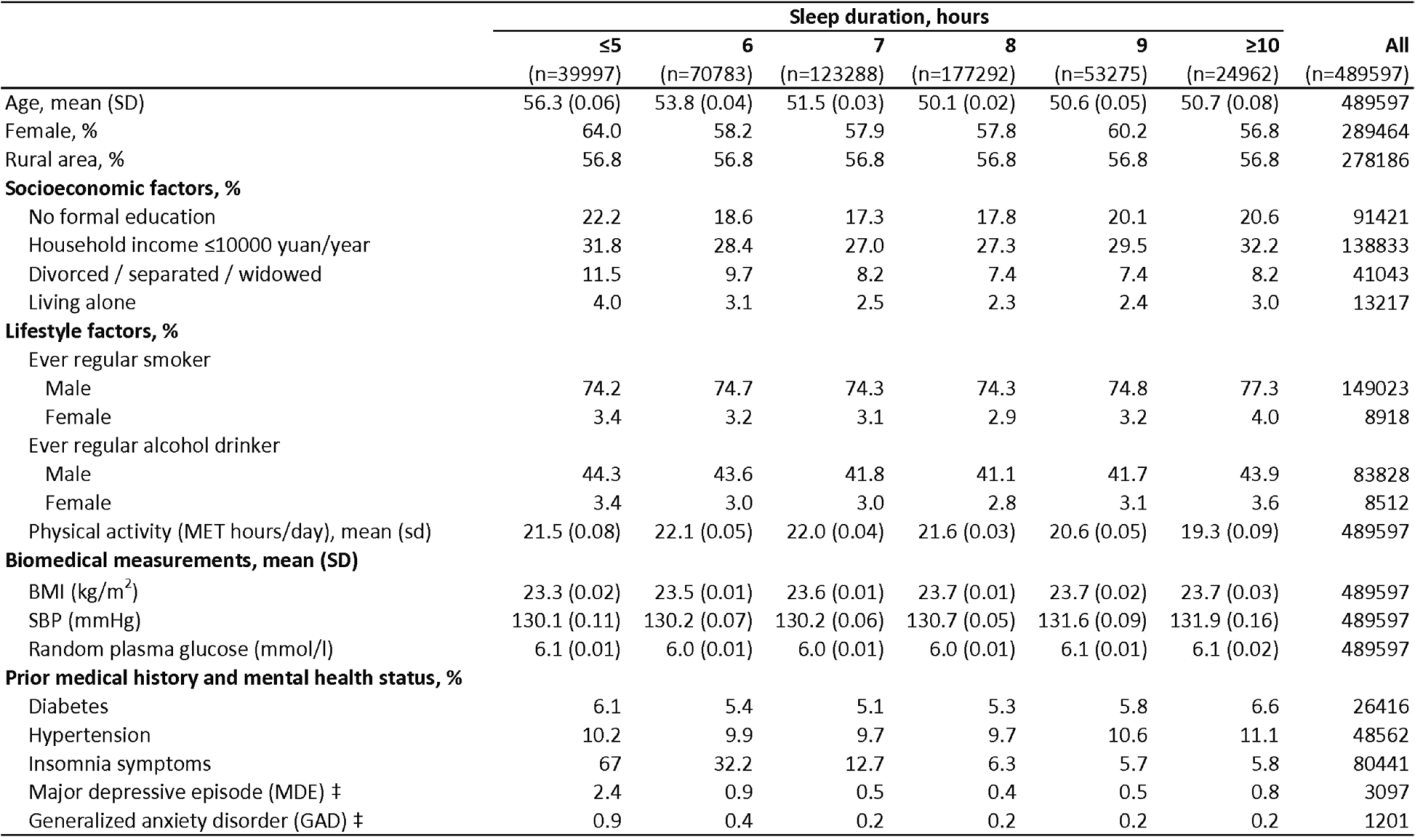
Baseline characteristics by sleep duration

**Fig. 1:**
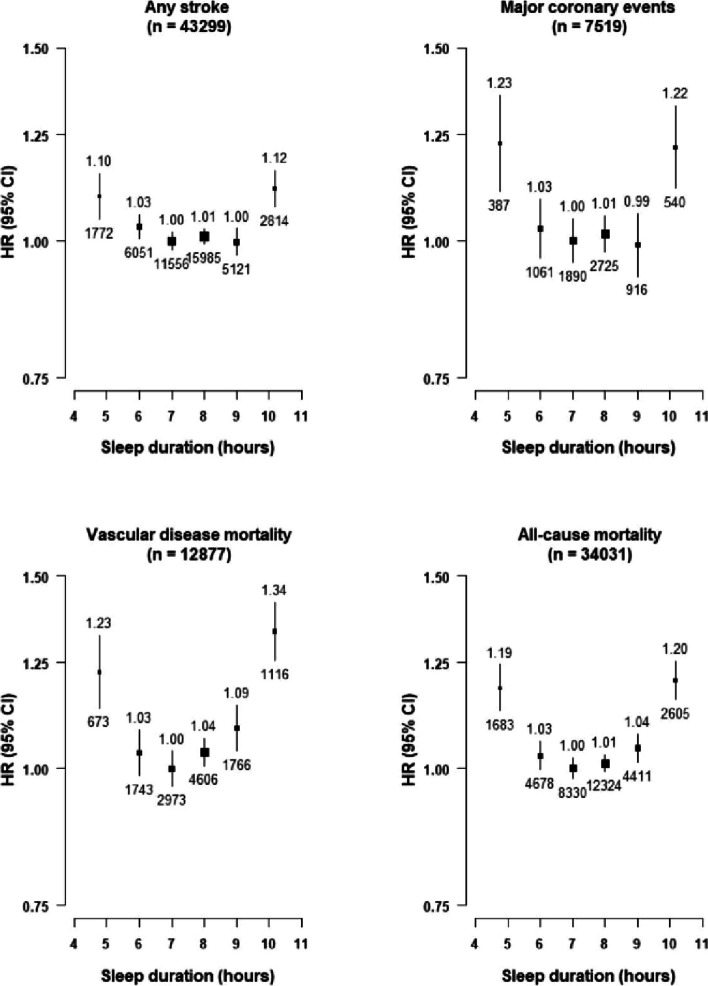
Adjusted Hazard Ratios (HRs) of any stroke, major coronary events, vascular mortality and all-cause mortality by self-reported sleep duration. Values shown above and below the squares are the adjusted HR and number of events, respectively. The size of the squares is proportional to ‘floating’ variance of the log HR in that group. The vertical lines represent the 95% CI. Models were stratified by region (10 regions) and adjusted for age at baseline (continuous), sex, smoking (never regular, ex-regular, or ever regular), alcohol intake (never regular drinker, ex regular drinker, occasional or seasonal drinker, monthly drinker, reduced intake, weekly), BMI (continuous, kg/m^2^), physical activity (Metabolic Equivalent of Task [MET] hours/day, continuous), systolic blood pressure (SBP) (continuous), SBP^2^, depression symptoms (yes/no), generalised anxiety symptoms (yes/no), snoring (yes, frequently; yes, sometimes; no/don't know), daytime napping (yes, yes but only in the summer, no). Age was used as the underlying time scale with entry at age at baseline

After 9 years of follow-up, a total of 43,215 (8.8%) participants died, including 16,378 (3.3%) deaths due to CVD. Moreover, 43,299 participants suffered a stroke, including 36,539 IS and 8128 ICH and 7519 had MCE events. Overall, sleep duration showed U-shaped associations with risks of stroke, MCE, vascular mortality and all-cause mortality, with those who reported sleep duration of 7–8 h having the lowest risks (Fig. 1). Compared with those who slept on average for 7–8 h, those with a very short sleep duration ≤ 5 h had an adjusted HR of 1.10 (95% CI 1.05–1.15) for stroke, 1.23 (1.11–1.36) for MCE, 1.34 (1.26–1.42) for vascular disease mortality and 1.20 (1.16–1.25) for all-cause mortality. Likewise, those who had a very long sleep duration ≥ 10 h had adjusted HRs of 1.12 (1.07–1.16) for stroke, 1.22 (1.12–1.33) for major coronary events, 1.34 (1.26–1.42) for vascular mortality, and 1.20 (1.16–1.25) for all-cause mortality (Table 2).

**Table 2:**
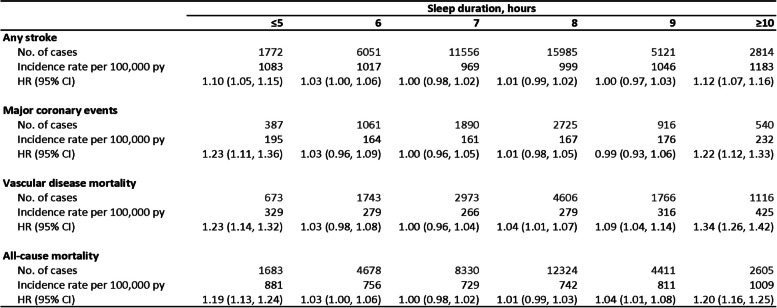
Adjusted HRs of CVD outcomes by sleep duration among individuals without prior CHD, stroke/TIA, or insomnia symptoms

**Fig. 2:**
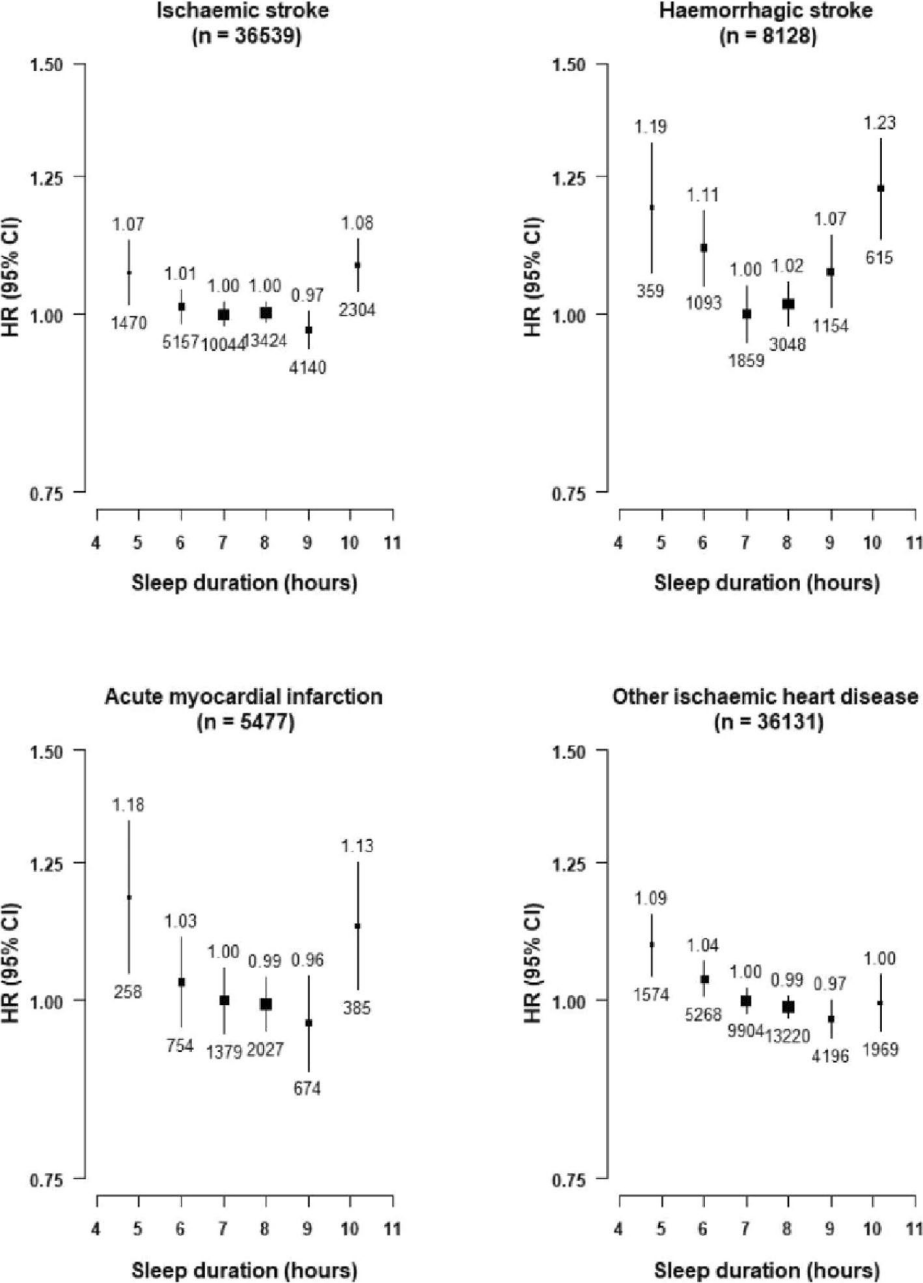
Adjusted Hazard Ratios (HRs) of ischaemic stroke (IS), Intracerebral Haemorrhage (ICH), Myocardial Infarction (MI) and other ischaemic Heart Diseases (Other IHD) by self-reported sleep duration. Each square represents an adjusted HR and the size of the squares is inversely proportional to the ‘floating’ variance of the log HR in that group. The horizontal lines represent the 95% CI. Models were stratified by region (10 regions) and adjusted for age at baseline (continuous), sex, smoking (never regular, ex-regular, or ever regular), alcohol intake (never regular drinker, ex regular drinker, occasional or seasonal drinker, monthly drinker, reduced intake, weekly), BMI (continuous, kg/m^2^), physical activity (Metabolic Equivalent of Task [MET] hours/day, continuous), systolic blood pressure (SBP) (continuous), SBP^2^, depression symptoms (yes/no), generalised anxiety symptoms (yes/no), snoring (yes, frequently; yes, sometimes; no/don't know), daytime napping (yes, yes but only in the summer, no). Age was used as the underlying time scale with entry at age at baseline. Symbols and conventions as in Fig. 1

Likewise, the associations of sleep duration with major stroke types were also U-shaped, with HRs associated with very short or very long sleep duration being somewhat more extreme for ICH (very short 1.19 [1.06–1.33], very long 1.23 [1.12–1.35] than for IS (1.07 [1.01–1.13], 1.08 [1.03–1.14]) (Fig. 2). For IHD types, the U-shaped associations were only evident for MI, but not for other (non-MI) IHD **(**Fig. 2**)**. The U-shaped associations with any stroke (eFigure 1), MCE (eFigure 2), vascular mortality (eFigure 3) and all-cause mortality (eFigure 4) were similar in men and women, and between individuals living in rural and in urban areas.

In sensitivity analyses, the shape and strength of the associations were unaltered after excluding individuals with any prior disease, poor self-rated health or events occurring during the first three-year of follow-up and after stepwise adjustment of age, sex, region, smoking, alcohol consumption, physical activity, BMI and systolic blood pressure and 12-month Major Depression Episode (MDE) and Generalised Anxiety Disorder (GAD) (eTable 1). The U-shaped associations were also present for individuals with or without hypertension and those with or without diabetes for any stroke (eFigure 5), MCE (eFigure 6), vascular mortality (eFigure 7) and all-cause mortality (eFigure 8). The U-shaped associations of sleep duration with major CVD types were unaltered by excluding individuals with prior diseases at baseline (eFigure 9), or disease outcomes occurring during the first 3 years of follow up (eFigure 10), or individuals with poor self-rated health (eFigure 11), or those who reported daytime napping (eFigure 12; for vascular mortality only). All of these exclusions had little effect on the shape or strength of the associations of sleep duration for any stroke, MCE, vascular mortality or all-cause mortality, respectively.

**Fig. 3:**
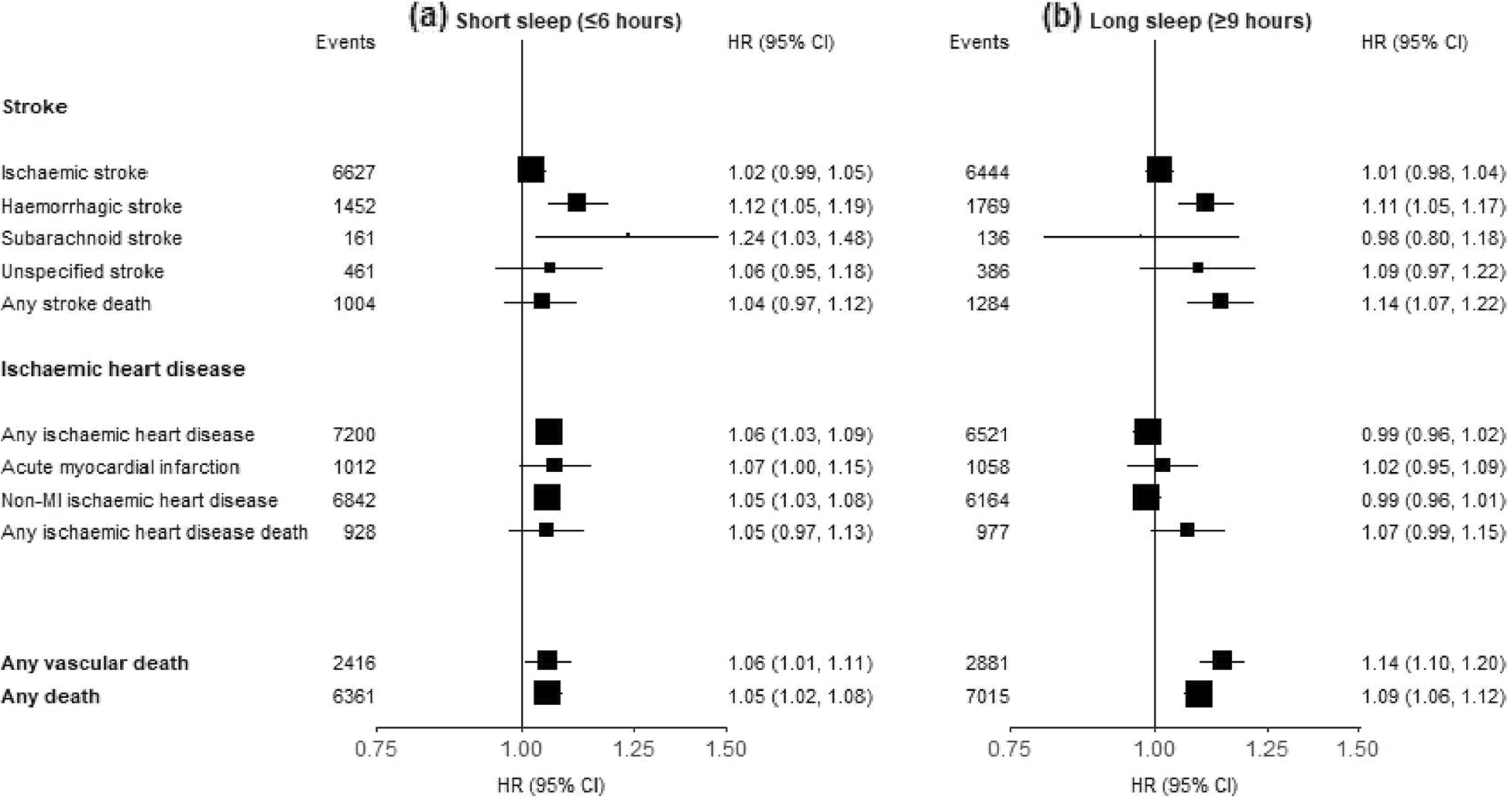
Adjusted Hazard Ratios (HRs) of fatal and non-fatal CVD outcomes for short (≤ 6 h) and long (≥ 9 h) sleep duration compared to sleep duration of 7–8 h). Each square represents an adjusted HR and the size of the squares is inversely proportional to the ‘floating’ variance of the log HR in that group. The vertical lines represent the 95% CI. Models were stratified by region (10 regions) and adjusted for age at baseline (continuous), sex, smoking (never regular, ex-regular, or ever regular), alcohol intake (never regular drinker, ex regular drinker, occasional or seasonal drinker, monthly drinker, reduced intake, weekly), BMI (continuous, kg/m^2^), physical activity (Metabolic Equivalent of Task [MET] hours/day, continuous), systolic blood pressure (SBP) (continuous), SBP^2^, depression symptoms (yes/no), generalised anxiety symptoms (yes/no), snoring (yes, frequently; yes, sometimes; no/don't know), daytime napping (yes, yes but only in the summer, no). Age was used as the underlying time scale with entry at age at baseline

Additional investigations examined the associations of the very short (≤ 6 h) vs long (≥ 9 h) sleep duration with major CVD types. For non-fatal stroke, both short and long sleep duration, were associated with, respectively, 12% (1.12; 1.05–1.19) and 11% (1.11; 1.05–1.17) higher risks of ICH, but with only 2% (1.02; 0.99–1.05) and 1% (1.01; 0.98–1.04) higher risk of IS (Fig. 3). For stroke death, only long sleep duration, but not short sleep duration, was associated with 14% (1.14; 1.07–1.22) higher risk (Fig. 3). For non-fatal IHD outcomes only short sleep duration, but not long sleep duration (Fig. 3), were associated with higher risks of non-fatal IHD and non-MI IHD events. For fatal outcomes, long sleep duration was associated with a 14% higher risk of vascular death and a 9% higher risk of death from any cause (Fig. 3).

Overall, short sleep duration (≤ 6 h) was associated with 7% (1.07; 1.05–1.09) higher risk of any incident stroke, particularly, in individuals aged less than 55 years and those with higher levels of education (eFigure 13). Short sleep duration was also associated with a 9% higher risks for MCE and vascular mortality (eFigure 14) and these associations were consistent in subgroups classified by age, sex, education or life-style factors. Overall, long sleep duration (≥ 9 h) was associated with 5% higher risk of any incident stroke, particularly, in males and those with low level of physical activity (MET < 15 h per day) (eFigure 13). Long sleep duration was also associated with 9% higher risks for MCE particularly for individuals living in urban areas and those had higher level of education (eFigure 14). Overall long sleep duration was associated with 16% (1.16; 1.11–1.21) higher risk of CVD particularly among those living in urban areas (1.26; 1.16–1.38; heterogeneity test; *p* ≤ 0.01; eFigure 15).

## Discussion

This large study involving over 0.5 million middle-aged Chinese adults with no prior CVD and insomnia, showed U-shaped associations of sleep duration with incident risks of major CVD types. Compared with those who had on average sleep duration of 7–8 h of sleep, both short (≤ 6 h) and long (≥ 9 h) sleep duration, particularly, very short (≤ 5 h) and very long (≥ 10 h) sleep duration, had significantly higher risks of incident CVD and CVD mortality. The U-shaped associations were more extreme for stroke, especially ICH, than for IHD. Moreover, the adverse effects of short sleep duration with IHD were more extreme in individuals living in urban than in rural areas and in those with higher levels of education. In contrast, the adverse effects of long sleep duration with stroke (particularly ICH) were more extreme in men than women and in those living in rural than urban areas and among current than non-smokers. The study demonstrated that the U-shaped associations of sleep duration with CVD mortality were unaltered by the presence or absence of daytime napping or seasonal differences in napping.

In recent decades, the associations of sleep duration with risks of major CVD types have attracted considerable interest. A meta-analysis of 15 studies, involving 474,684 individuals and 4169 IHD outcomes and 3478 stroke outcomes, reported U-shaped associations of sleep duration with risks of CVD [3]. Similar findings have been reported in a meta-analysis of 67 prospective studies, involving over 3.5 million individuals with 22,511 IHD, 15,476 stroke cases, and 241,107 deaths from any cause [2]. However, a recent prospective study, involving 12 805 adults aged 17–80 years [11] from a multi-ethnic cohort, reported that short sleep duration was unrelated to risk of CVD in Asian populations, with exception of individuals with obesity and diabetes. However, most previous meta-analysis and individual cohort studies [2, 12, 13], did not assess associations of sleep duration with CVD subtypes. Moreover, previous studies typically used short sleep duration (≤ 6 h) or long sleep duration (≥ 9 h) and did not exclude individuals with insomnia symptoms. Insomnia symptoms are characterised as difficulty in falling asleep, staying awake, or experiencing non-restorative sleep, which results in inadequate sleep duration particularly short sleep duration. Including individuals with insomnia in sleep duration studies could introduce confounding that may obscure the true associations of sleep duration with CVD outcomes. Moreover, insomnia may also have independent effects on CVD outcomes and may be associated with multiple different physiological and psychological mechanisms compared with individuals without insomnia. Hence, it is important, to study participants with insomnia symptoms separately from other individuals when investigating the effects of insomnia symptoms on cardiovascular disease outcomes. Overall, excluding insomnia patients from sleep duration studies should provide more reliable evidence about the shape and strength of associations of sleep duration with CVD outcomes.

A prospective analysis of CKB, including 9,692 stroke-free participants, aged 42–81 years from the European Prospective Investigation into Cancer–Norfolk cohort, included 346 stroke outcomes during 9.5 years of follow-up and suggested that short sleep (≤ 6 h) duration was associated with higher risks of ischemic stroke, but long sleep duration (≥ 9 h) was associated with higher risks of hemorrhagic stroke [14]. A Korean case–control study, involving 490 stroke cases, also reported that long sleep duration was associated with higher risks of hemorrhagic stroke [15]. In contrast, the present study demonstrated that both short and long sleep duration were more strongly associated with hemorrhagic stroke than with ischemic stroke. Moreover, compared to short sleep duration, long sleep duration was associated with higher risks of stroke, consistent with the recent meta-analysis of prospective studies of stroke [12] involving 528,653 participants and 12,193 fatal and non-fatal stroke events.

Overall, the U-shaped associations were similar in subgroups classified by age, sex, region, levels of BMI or MET, prior non-CVD or mental disorders, consistent with a recent meta-analysis of previous studies mostly in Western populations [2]. However, the associations of short sleep duration with stroke were stronger in younger than in older people (< 55 years), whilst those for long sleep duration with stroke were stronger in men than in women.

### Strengths and limitations

The chief strengths of the present study included the large sample size, and detailed data on confounders, including a wide range demographic, socio-economic, lifestyle factors and information on prior mental and physical illnesses in a single study. The number of CVD outcomes and the large sample size of the study enabled detailed analyses of major CVD types overall and in relevant subgroups of participants. Moreover, the present study used sleep duration in hours rather than categories (short vs long) and excluded individuals with insomnia symptoms, to minimise the effects of reverse causality. However, the present study also had several limitations. Firstly, napping which was included in the sleep duration could confound the associations of sleep duration with CVD risk although the association was not altered in the sensitivity analysis with and without napping. Secondly, sleep apnoea was not measured and daytime napping was not recorded separately from sleep duration in the present study, which could confound the associations of sleep duration with risk of CVD. The associations of sleep apnoea and daytime napping with CVD risk should be examined independently of sleep duration in future studies. Consistent with other large cohorts, the present study used self-reported sleep duration, in which measurement error cannot be fully excluded. Future studies should include objective measurements to verify self-reported measurements of sleep duration. Despite excluding major depressive disorders, insomnia symptoms, prior CVD events or events occurring during the first 3 years of follow-up, the possibility of residual confounding or reverse causation cannot be fully excluded.

Despite the associations of short and long sleep duration with higher risks of CVD observed in Chinese population consistent with the findings reported in Western populations, the mechanisms underlying these associations remain uncertain. However, the present study was unable to determine the mechanisms underlying these associations of sleep duration with CVD outcomes. It is possible that short sleep duration may lead result in alternation of several physiological processes, including blood pressure, inflammation, glucose metabolism or hormone secretion which could contribute the development of cardiovascular diseases outcomes.

## Conclusions

This prospective study of Chinese adults demonstrated U-shaped associations of abnormal sleep duration with both stroke types and IHD types with no prior vascular disease. Compared with an average sleep duration of 7–8 h, individuals with a short (≤ 6 h) or long (≥ 9 h) sleep duration had higher risks of both fatal and non-fatal CVD outcomes. However, these associations were much more extreme in those with very short (≤ 5 h) or very long (≥ 10 h) sleep duration. Understanding the relevance of these associations and targeting individuals with very abnormal sleep duration for more intensive treatment have implications for prevention of major CVD types.

### Supplementary Information

**Additional file 1.**

## Data Availability

The China Kadoorie Biobank (CKB) is a global resource for the investigation of lifestyle, environmental, blood biochemical and genetic factors as determinants of common diseases. The CKB study group is committed to making the cohort data available to the scientific community in China, the UK and worldwide to advance knowledge about the causes, prevention and treatment of disease. For detailed information on what data is currently available to open access users and how to apply for it, visit: http://www.ckbiobank.org/site/Data+Access. Bona fide researchers who wish to obtain the individual level data from the China Kadoorie Biobank study included in this report should contact ckbaccess@ndph.ox.ac.uk. A research proposal will be requested to ensure that any analyses are performed by appropriate researchers and—where data is not currently available to open access researchers—are restricted to the topic of this paper.

## Abbreviations

BMI: Body mass index
CI: Confidence interval
CVD: Cardiovascular disease
HI: Health insurance
HR: Hazard ratio
ICH: Intracerebral haemorrhage
IHD: Ischaemic heart disease
IS: Ischaemic stroke
MET: Metabolic Equivalent of Task
MI: Myocardial infarction
SBP: Systolic blood pressure
