# Supplementary material for: Sleep duration and risk of stroke and coronary heart disease: a 9-year community-based prospective study of 0.5 million Chinese adults
Source: BMC Neurol. Author manuscript; Available in PMC 2023 Sep 26. (PMC10500908; doi:10.1186/s12883-023-03367-4)

Supplementary material for

Sleep duration and risk of stroke and coronary heart disease: a 9-year community-based prospective study of 0.5 million Chinese adults

**Table of Contents**

[Members of the China Kadoorie Biobank Collaborative Study Group 2](#_Toc112917639)

[ICD-10 Codes used for disease outcomes 3](#_Toc112917640)

[eTable1: Adjusted HRs of CVD outcomes by sleep duration among individuals without prior CVD, stroke/TIA or insomnia symptoms 4](#_Toc112917641)

[eFigure 1. HRs (95% CIs) of any stroke by sleep duration (hours), in subgroups by sex and urban/rural residence 5](#_Toc112917642)

[eFigure 2. HRs (95% CIs) of MCE by sleep duration (hours), in subgroups by sex and urban/rural residence 6](#_Toc112917643)

[eFigure 3. HRs (95% CIs) of vascular disease mortality by sleep duration (hours), in subgroups by sex and urban/rural residence 7](#_Toc112917644)

[eFigure 4. HRs (95% CIs) of all-cause mortality by sleep duration (hours), in subgroups by sex and urban/rural residence 8](#_Toc112917645)

[eFigure 5. HRs (95% CIs) of any stroke by sleep duration (hours), in subgroups by hypertension and diabetes at baseline 9](#_Toc112917646)

[eFigure 6. HRs (95% CIs) of major coronary events by sleep duration (hours), in subgroups by hypertension and diabetes at baseline 10](#_Toc112917647)

[eFigure 7. HRs (95% CIs) of vascular disease mortality by sleep duration (hours), in subgroups by hypertension and diabetes at baseline 11](#_Toc112917648)

[eFigure 8. HRs (95% CIs) of all-cause mortality by sleep duration (hours), in subgroups by hypertension and diabetes at baseline 12](#_Toc112917649)

[eFigure 9. Adjusted HRs of CVD outcomes by sleep duration, among individuals with no prior disease at baseline 13](#_Toc112917650)

[eFigure 10. Adjusted HRs of CVD outcomes by sleep duration, excluding the first three years of follow-up 14](#_Toc112917651)

[eFigure 11. Adjusted HRs of CVD outcomes by sleep duration, excluding individuals with poor self-related health 15](#_Toc112917652)

[eFigure 12. HRs (95% CIs) of vascular disease mortality by sleep duration (hours) in subgroups by daytime napping 16](#_Toc112917653)

[eFigure 13. Adjusted HRs for incident stroke for (a) short and (b) long sleep duration vs normal sleep duration 17](#_Toc112917654)

[eFigure 14. Adjusted HRs for major coronary events (MCE) for (a) short and (b) long vs normal sleep duration by subgroups 18](#_Toc112917655)

[eFigure 15. Adjusted HRs for vascular mortality for (a) short and (b) long sleep duration vs normal sleep duration, by subgroups 19](#_Toc112917656)

# ****Members of the China Kadoorie Biobank Collaborative Study Group****

International Steering Committee: Junshi Chen, Zhengming Chen (PI), Robert Clarke, Rory Collins, Yu Guo, Liming Li (PI), Jun Lv, Richard Peto, Robin Walters. International Co-ordinating Centre, Oxford: Daniel Avery, Ruth Boxall, Derrick Bennett, Yumei Chang, Yiping Chen, Zhengming Chen, Robert Clarke, Huaidong Du, Simon Gilbert, Alex Hacker, Mike Hill, Michael Holmes, Andri Iona, Christiana Kartsonaki, Rene Kerosi, Garry Lancaster, Sarah Lewington, Kuang Lin, John McDonnell, Iona Millwood, Qunhua Nie, Jayakrishnan Radhakrishnan, Paul Ryder, Sam Sansome, Dan Schmidt, Paul Sherliker, Rajani Sohoni, Becky Stevens, Iain Turnbull, Robin Walters, Jenny Wang, Lin Wang, Neil Wright, Ling Yang, Xiaoming Yang. National Co-ordinating Centre, Beijing: Yu Guo, Xiao Han, Can Hou, Jun Lv, Pei Pei, Chao Liu, Canqing Yu, Qingmei Xia. 10 Regional Co-ordinating Centres: Qingdao CDC: Zengchang Pang, Ruqin Gao, Shanpeng Li, Shaojie Wang, Yongmei Liu, Ranran Du, Yajing Zang, Liang Cheng, Xiaocao Tian, Hua Zhang, Yaoming Zhai, Feng Ning, Xiaohui Sun, Feifei Li. Licang CDC: Silu Lv, Junzheng Wang, Wei Hou. Heilongjiang Provincial CDC: Mingyuan Zou, Ge Jiang, Xue Zhou. Nangang CDC: Liqiu Yang, Hui He, Bo Yu, Yanjie Li, Qinai Xu, Quan Kang, Ziyan Guo. Hainan Provincial CDC: Dan Wang, Ximin Hu, Jinyan Chen, Yan Fu, Zhenwang Fu, Xiaohuan Wang. Meilan CDC: Min Weng, Zhendong Guo, Shukuan Wu, Yilei Li, Huimei Li, Zhifang Fu. Jiangsu Provincial CDC: Ming Wu, Yonglin Zhou, Jinyi Zhou, Ran Tao, Jie Yang, Jian Su. Suzhou CDC: Fang Liu, Jun Zhang, Yihe Hu, Yan Lu, Liangcai Ma, Aiyu Tang, Shuo Zhang, Jianrong Jin, Jingchao Liu. Guangxi Provincial CDC: Zhenzhu Tang, Naying Chen, Ying Huang. Liuzhou CDC: Mingqiang Li, Jinhuai Meng, Rong Pan, Qilian Jiang, Jian Lan,Yun Liu, Liuping Wei, Liyuan Zhou, Ningyu Chen Ping Wang, Fanwen Meng, Yulu Qin, Sisi Wang. Sichuan Provincial CDC: Xianping Wu, Ningmei Zhang, Xiaofang Chen, Weiwei Zhou. Pengzhou CDC: Guojin Luo, Jianguo Li, Xiaofang Chen, Xunfu Zhong, Jiaqiu Liu, Qiang Sun. Gansu Provincial CDC: Pengfei Ge, Xiaolan Ren, Caixia Dong. Maiji CDC: Hui Zhang, Enke Mao, Xiaoping Wang, Tao Wang, Xi zhang. Henan Provincial CDC: Ding Zhang, Gang Zhou, Shixian Feng, Liang Chang, Lei Fan. Huixian CDC: Yulian Gao, Tianyou He, Huarong Sun, Pan He, Chen Hu, Xukui Zhang, Huifang Wu. Zhejiang Provincial CDC: Min Yu, Ruying Hu, Hao Wang. Tongxiang CDC: Yijian Qian, Chunmei Wang, Kaixu Xie, Lingli Chen, Yidan Zhang, Dongxia Pan, Qijun Gu. Hunan Provincial CDC: Yuelong Huang, Biyun Chen, Li Yin, Huilin Liu, Zhongxi Fu, Qiaohua Xu. Liuyang CDC: Xin Xu, Hao Zhang, Huajun Long, Xianzhi Li, Libo Zhang, Zhe Qiu.

# ****ICD-10 Codes used for disease outcomes****


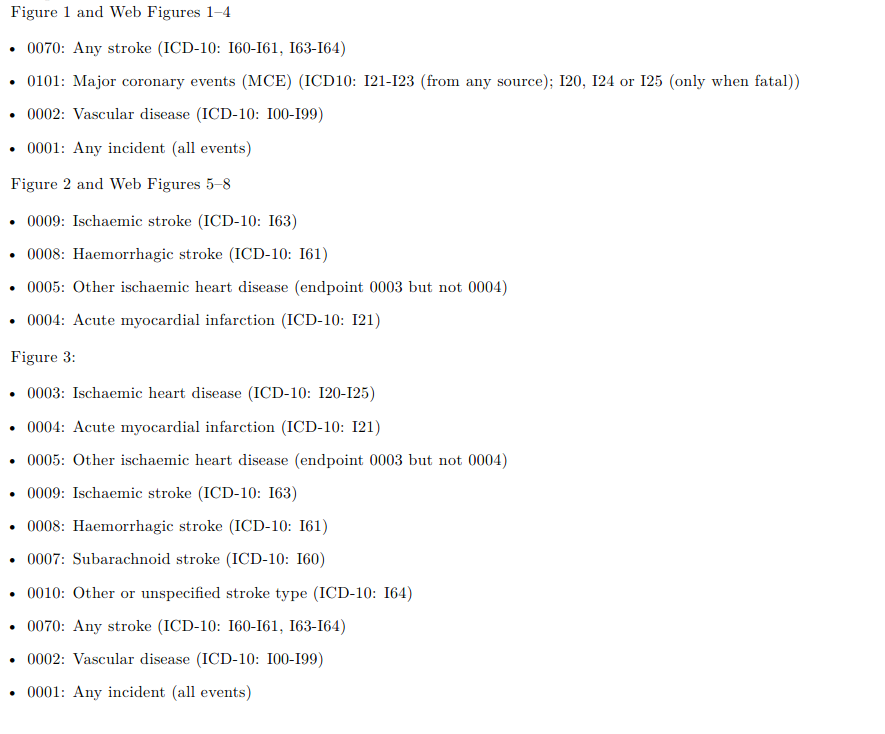


# ****eTable1: Adjusted HRs of CVD outcomes by sleep duration among individuals without prior CVD, stroke/TIA or insomnia symptoms****


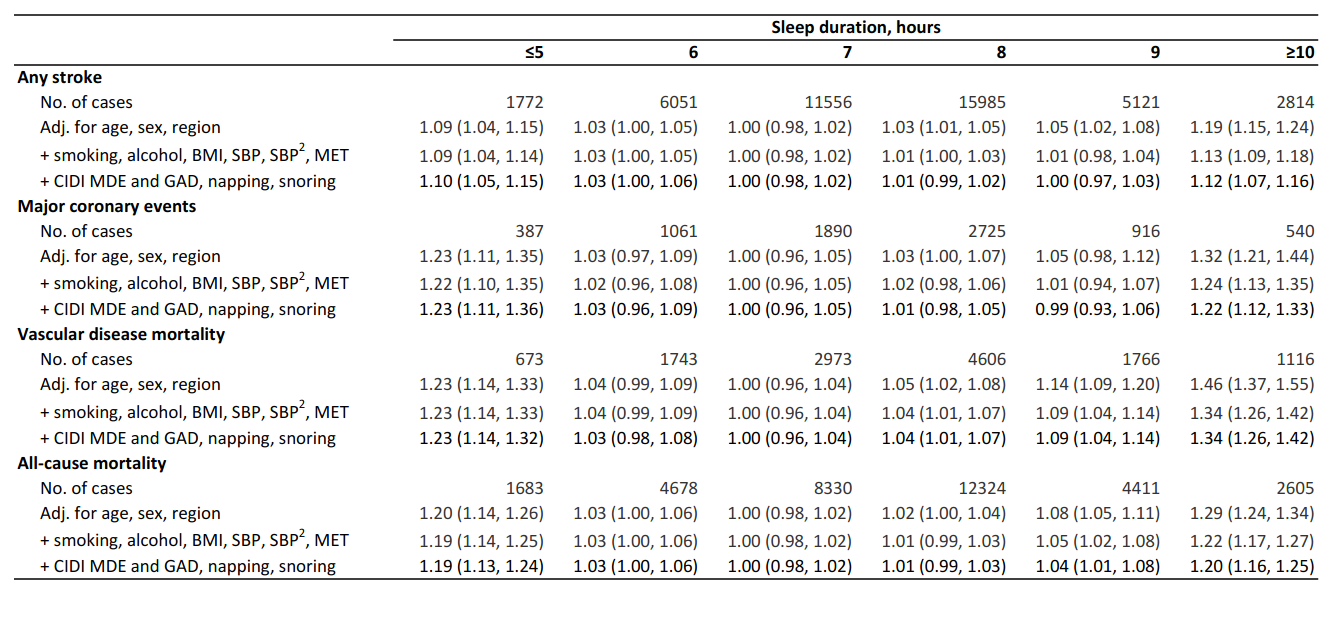


# ****eFigure 1. HRs (95% CIs) of any stroke by sleep duration (hours), in subgroups by sex and urban/rural residence****


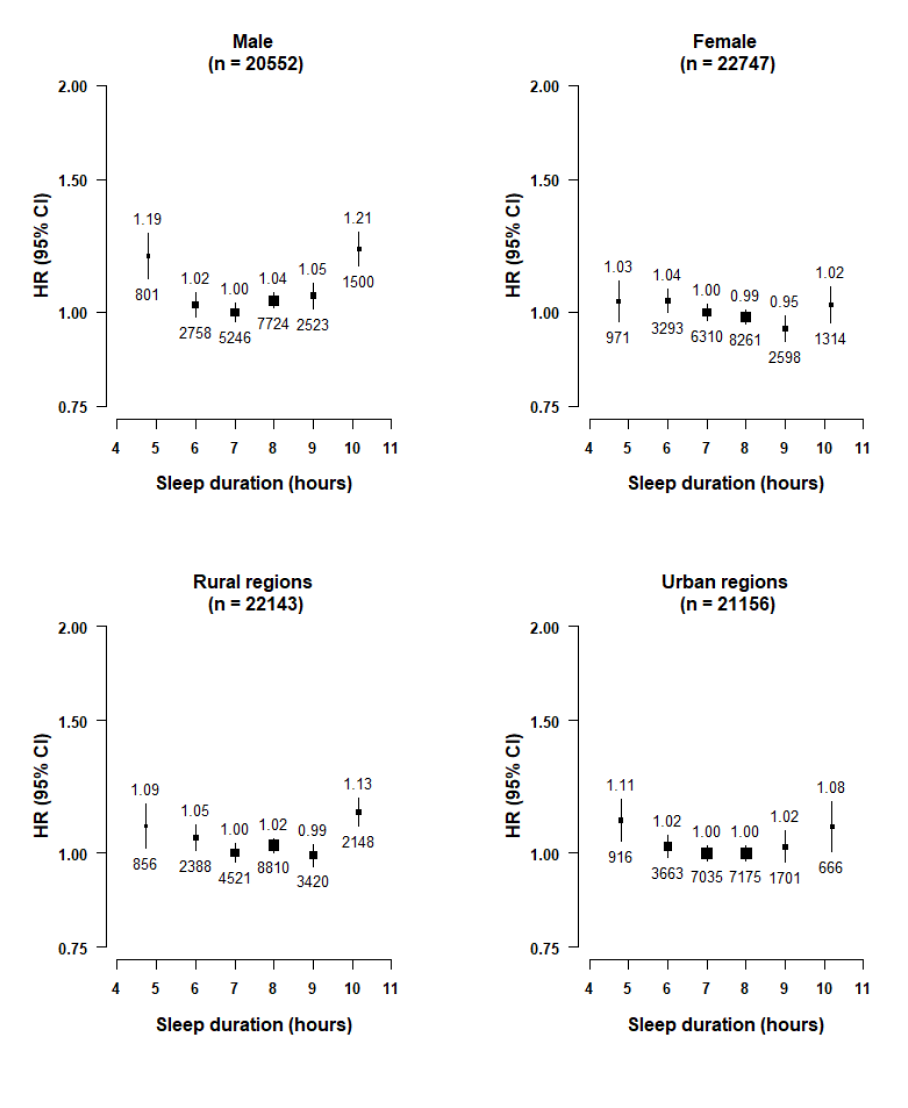


# ****eFigure 2. HRs (95% CIs) of MCE by sleep duration (hours), in subgroups by sex and urban/rural residence****


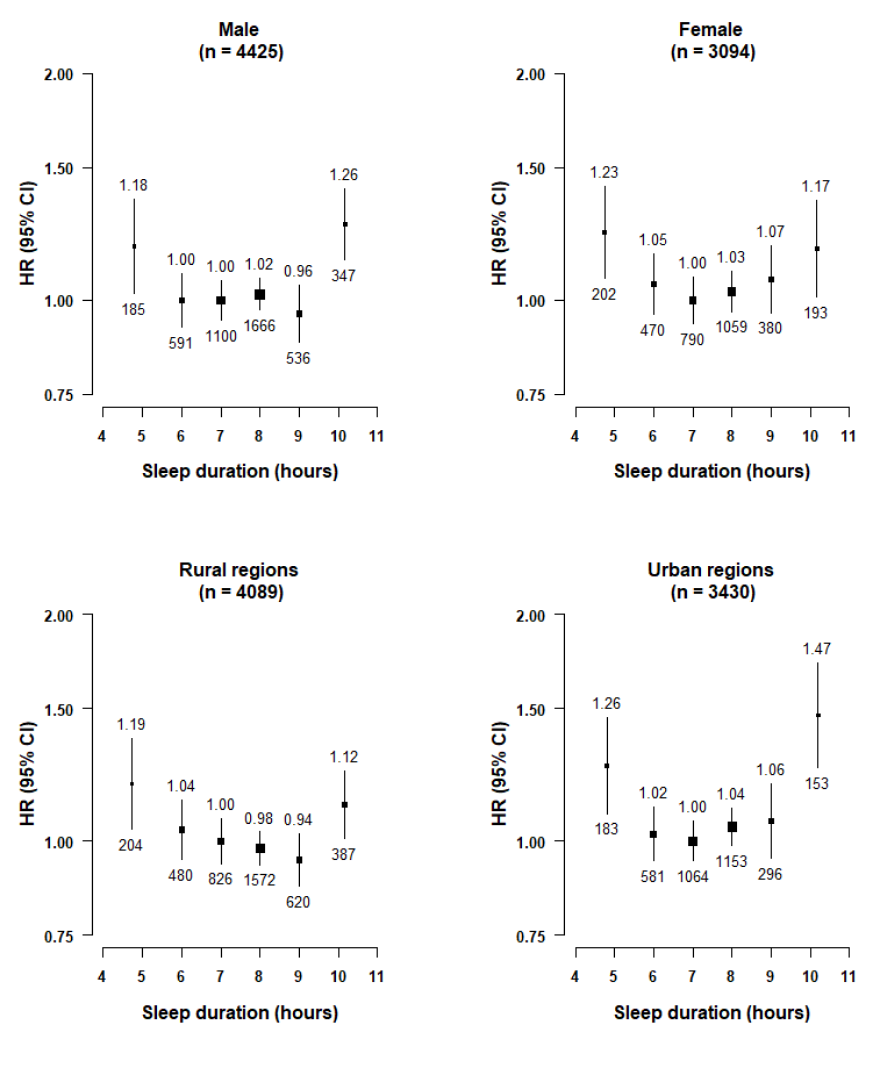


# ****eFigure 3. HRs (95% CIs) of vascular disease mortality by sleep duration (hours), in subgroups by sex and urban/rural residence****


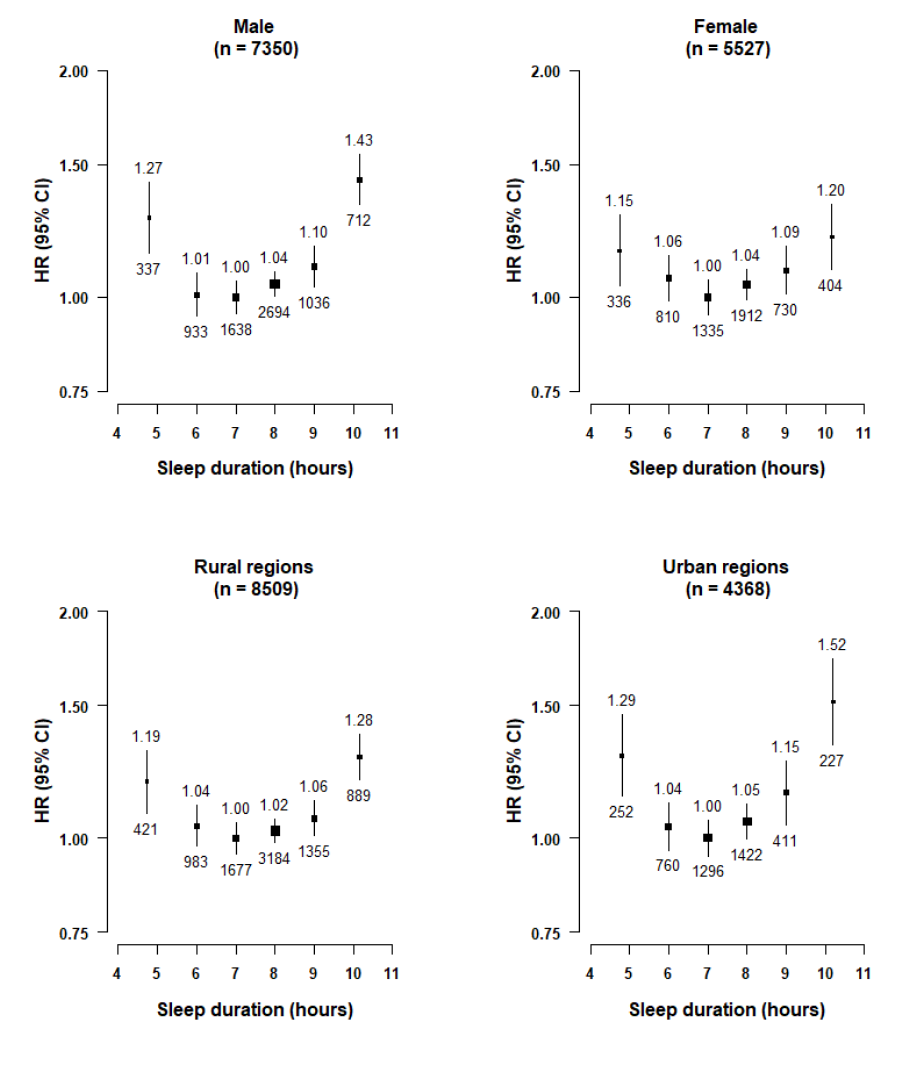


# ****eFigure 4. HRs (95% CIs) of all-cause mortality by sleep duration (hours), in subgroups by sex and urban/rural residence****


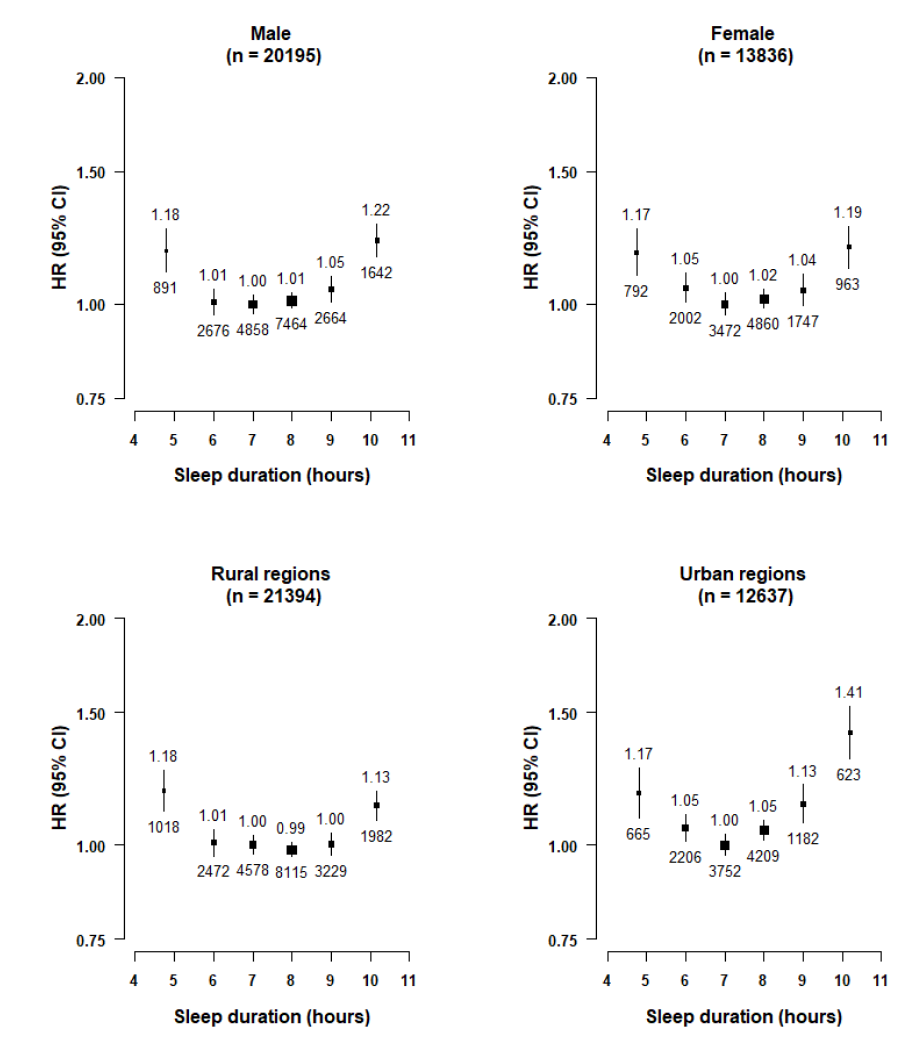


# ****eFigure 5. HRs (95% CIs) of any stroke by sleep duration (hours), in subgroups by hypertension and diabetes at baseline****


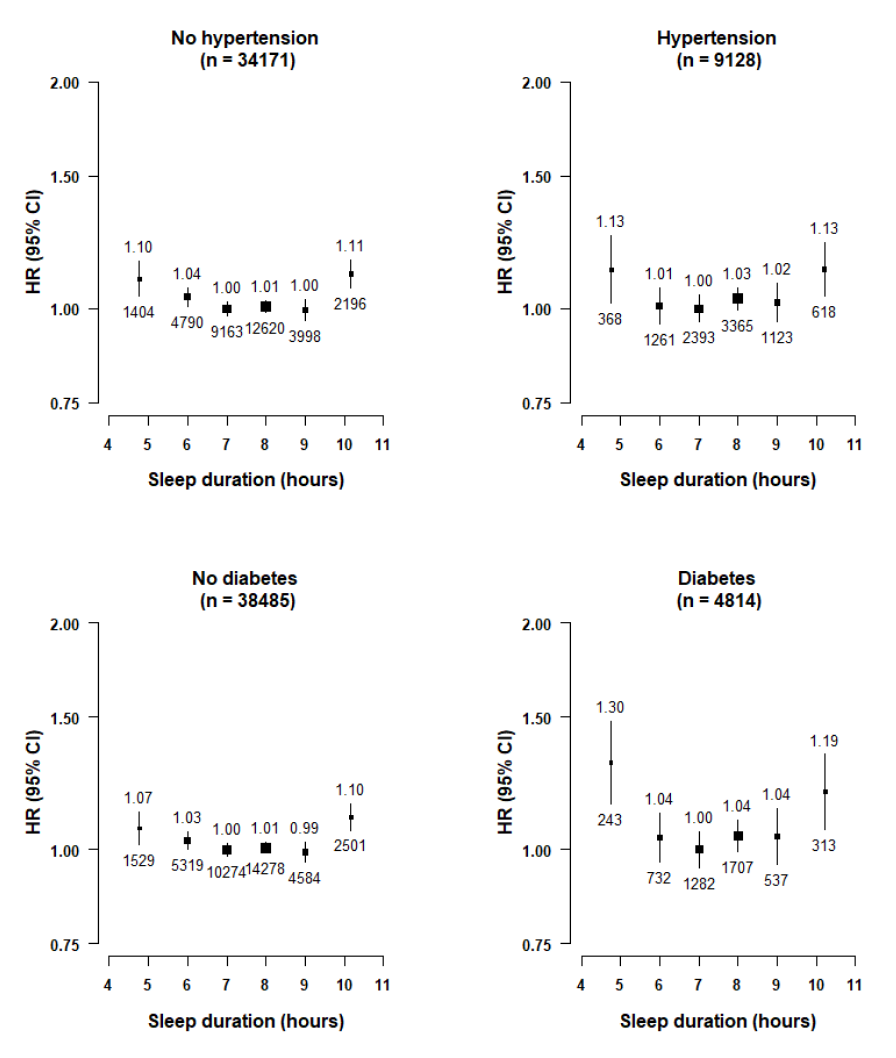


# ****eFigure 6. HRs (95% CIs) of major coronary events by sleep duration (hours), in subgroups by hypertension and diabetes at baseline****


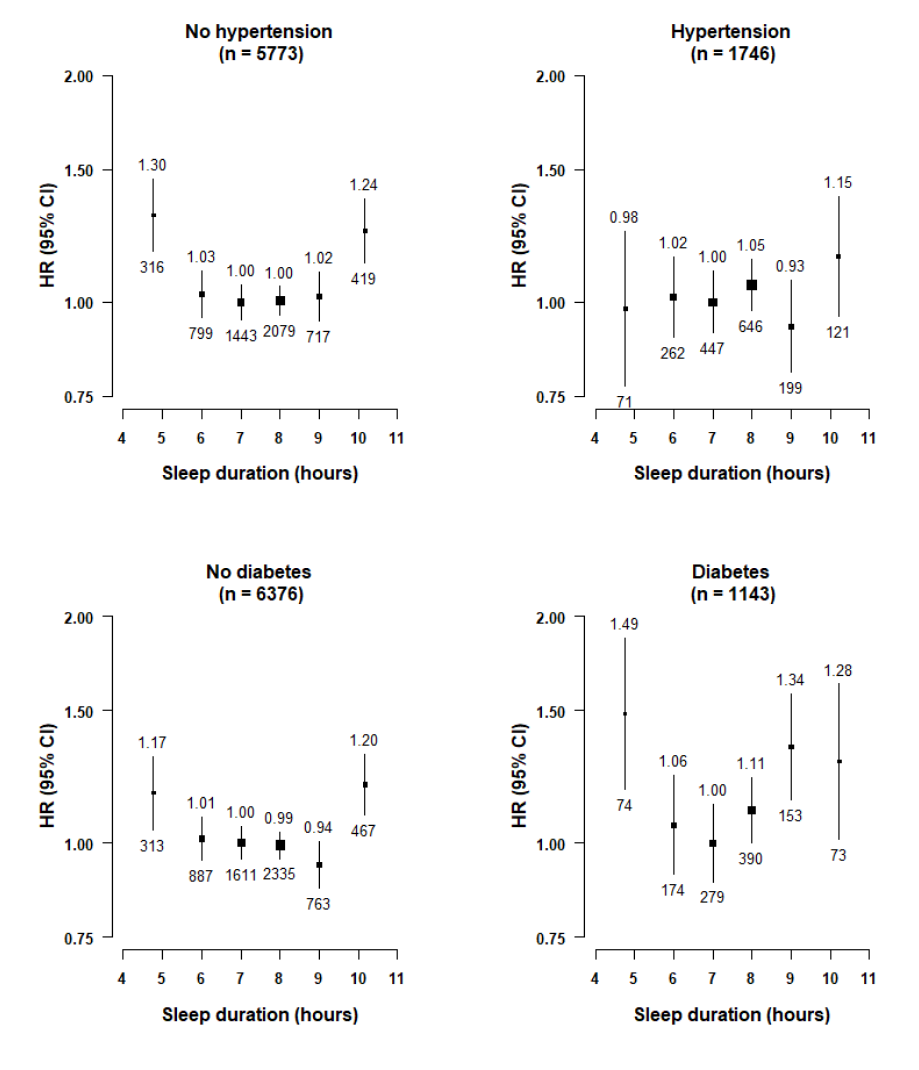


# ****eFigure 7. HRs (95% CIs) of vascular disease mortality by sleep duration (hours)****, ****in subgroups by hypertension and diabetes at baseline****


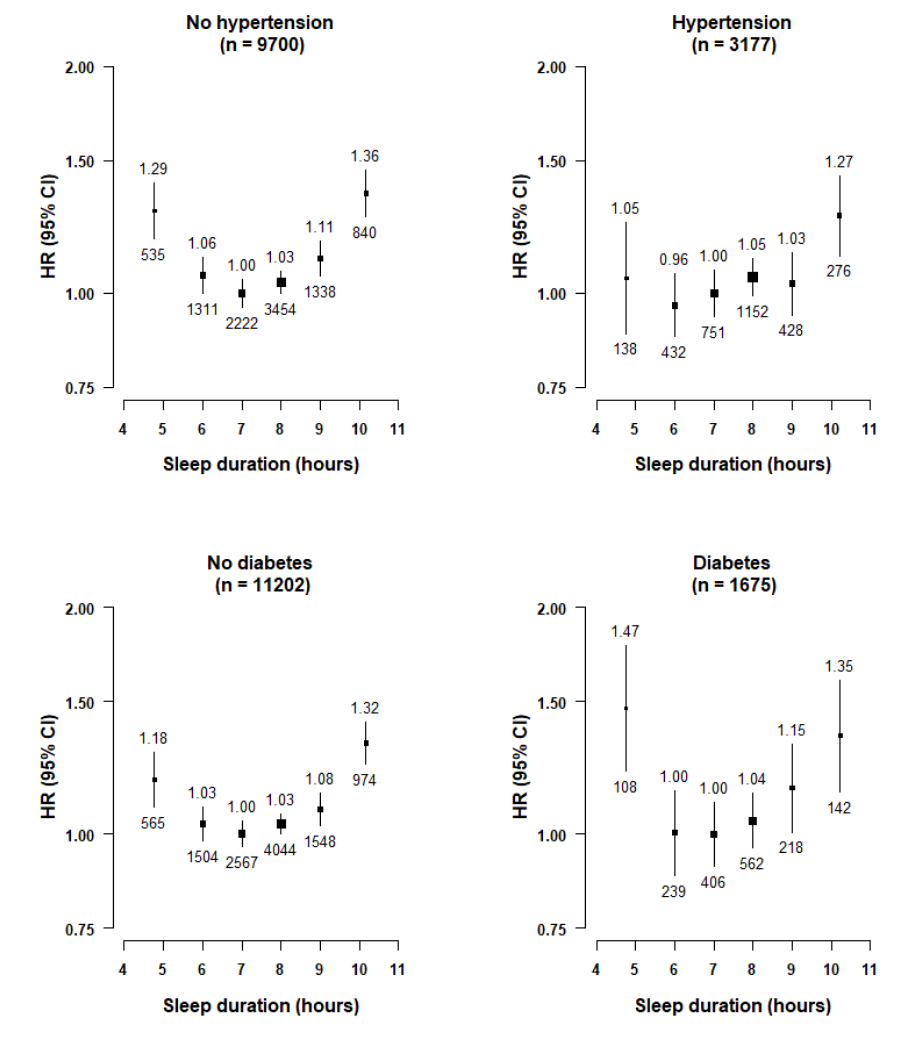


# ****eFigure 8. HRs (95% CIs) of all-cause mortality by sleep duration (hours), in subgroups by hypertension and diabetes at baseline****


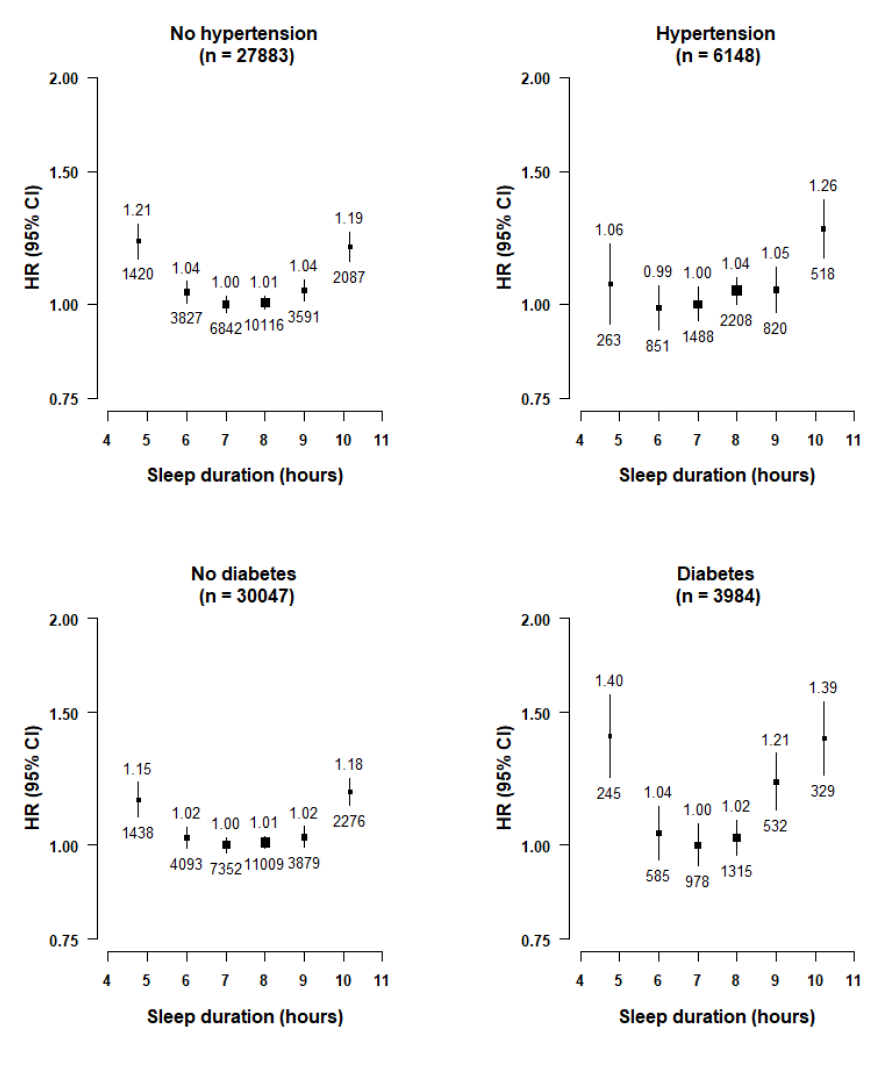


# ****eFigure 9. Adjusted HRs of CVD outcomes by sleep duration, among individuals with no prior disease at baseline****


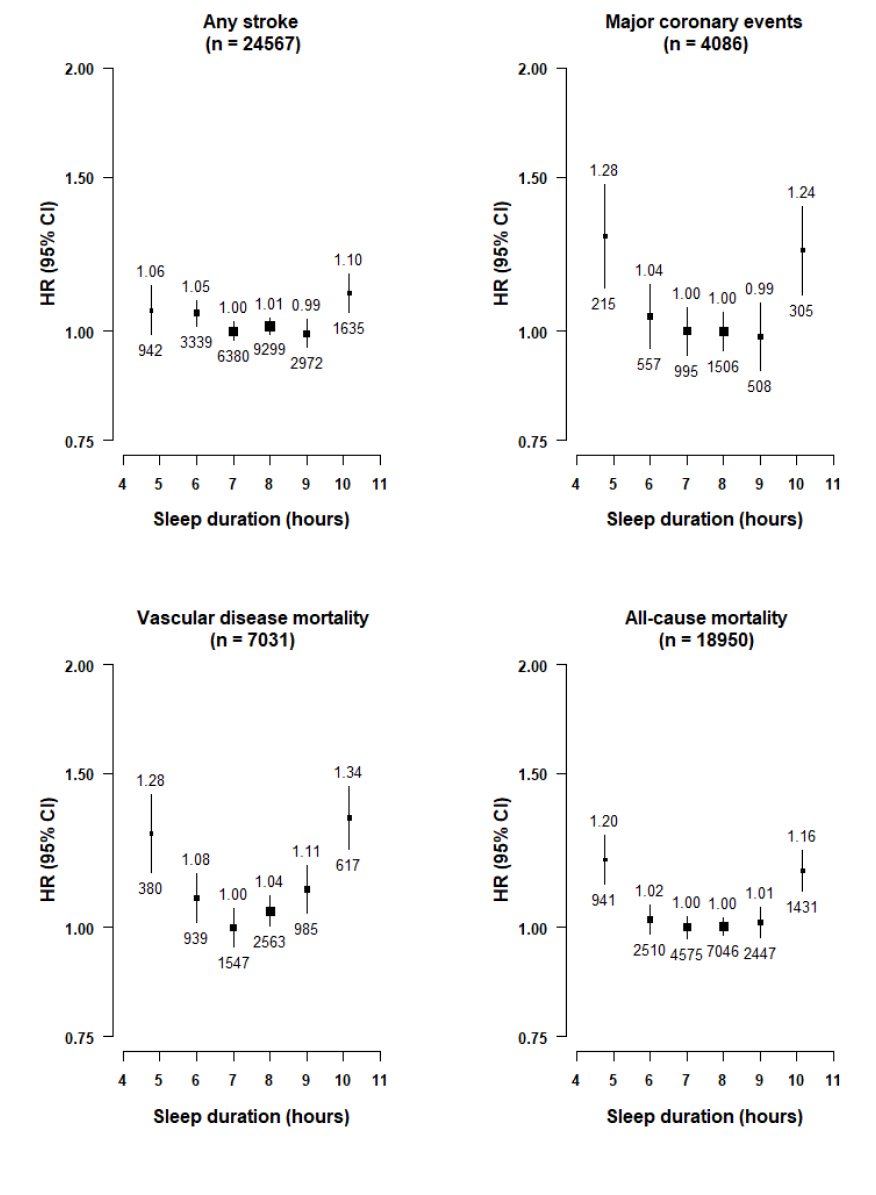


# ****eFigure 10. Adjusted HRs of CVD outcomes by sleep duration, excluding the first three years of follow-up****


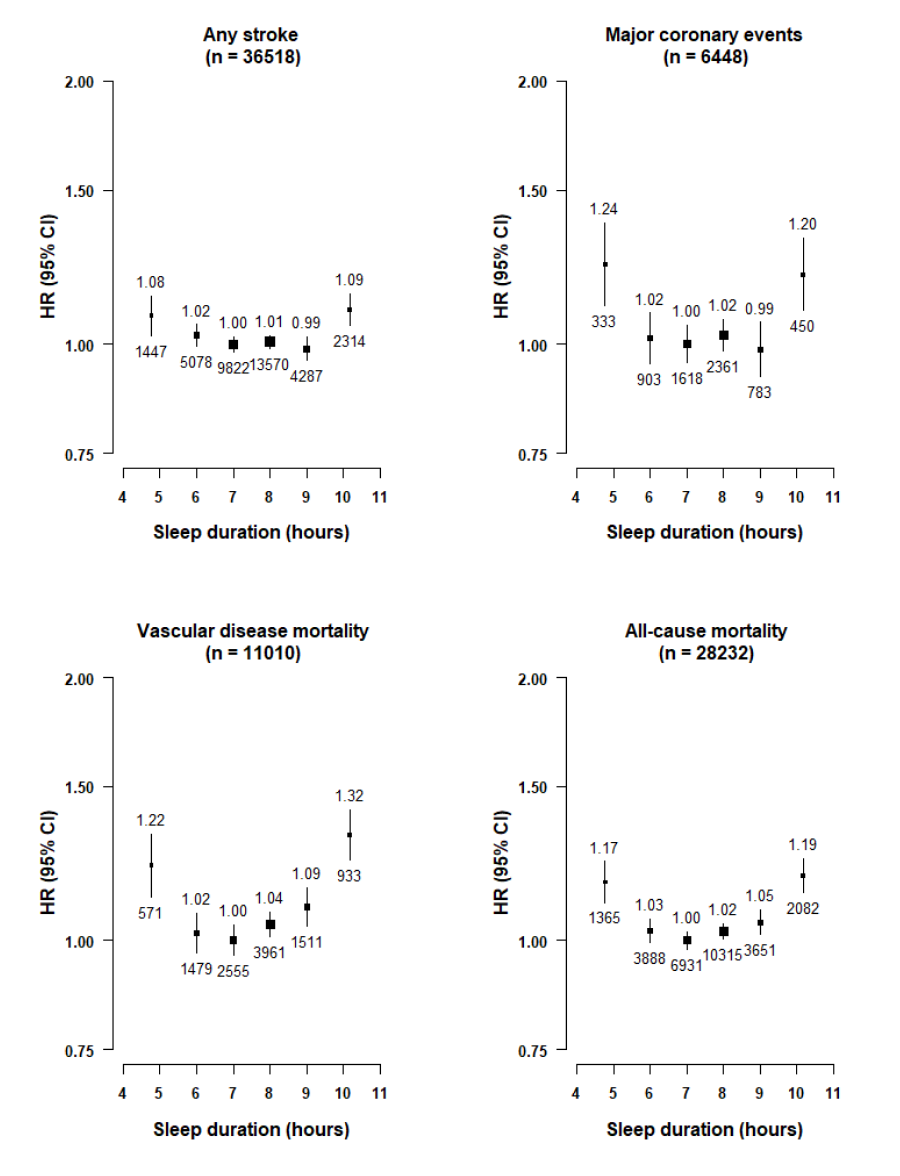


# ****eFigure 11. Adjusted HRs of CVD outcomes by sleep duration, excluding individuals with poor self-related health****


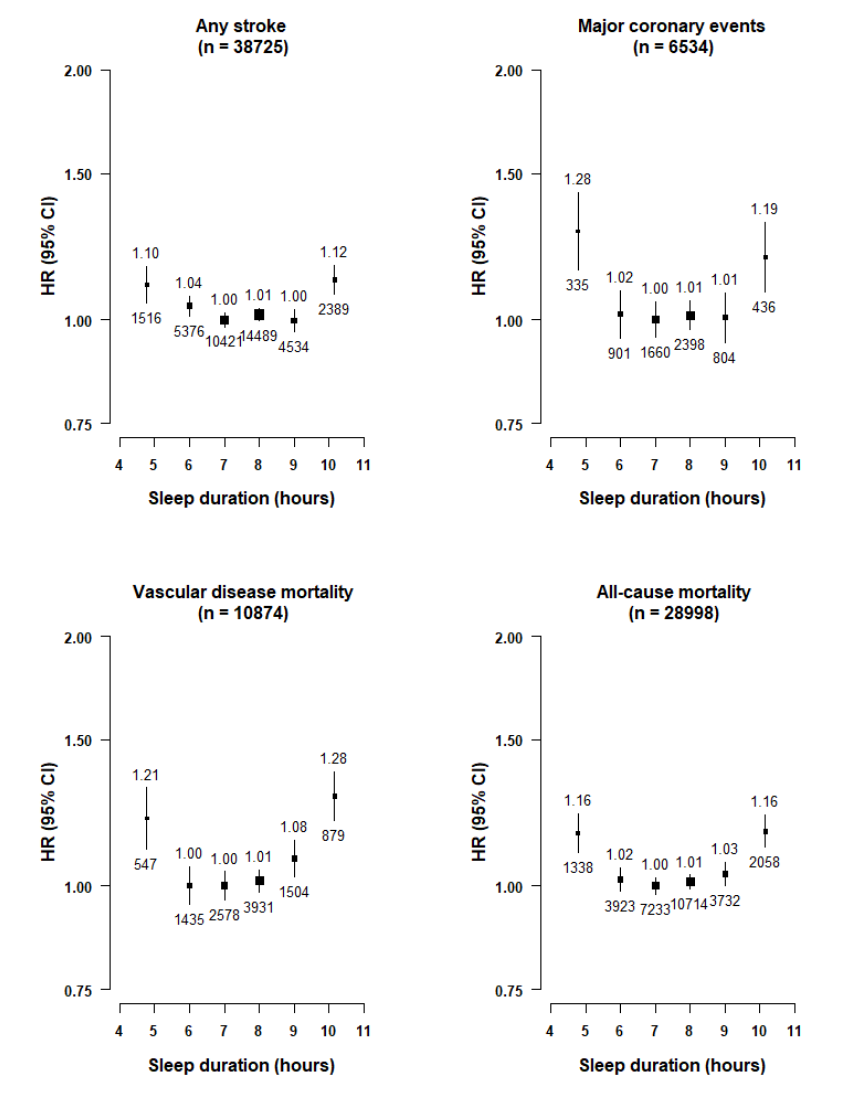


# ****eFigure 12. HRs (95% CIs) of vascular disease mortality by sleep duration (hours) in subgroups by daytime napping****


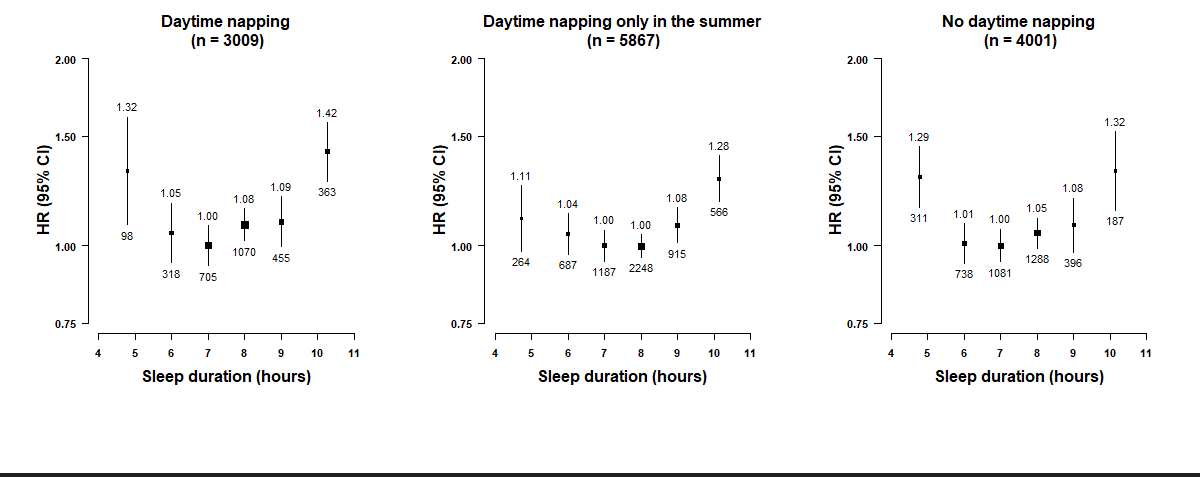


# ****eFigure 13. Adjusted HRs for incident stroke for (a) short and (b) long sleep duration vs normal sleep duration****


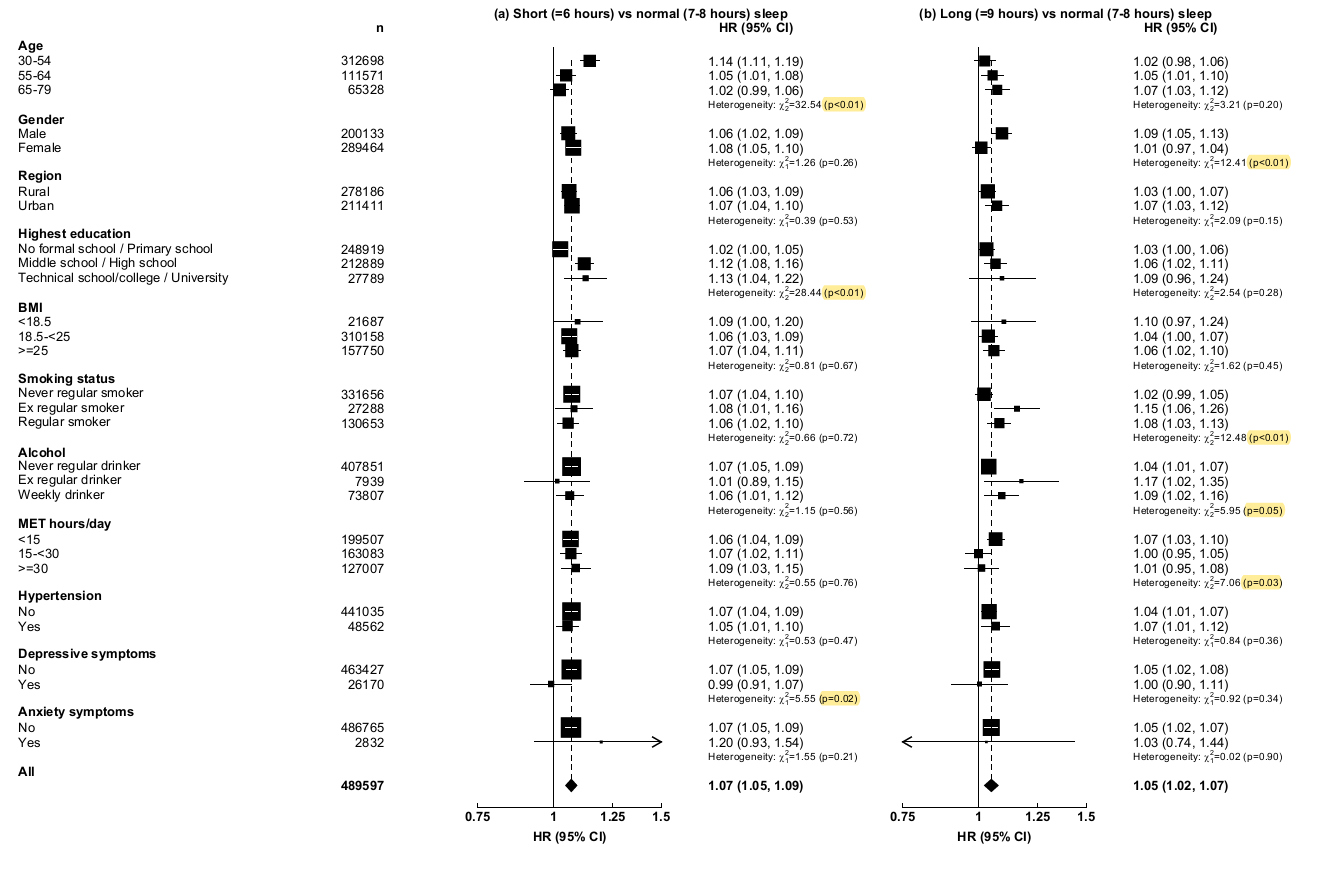


# ****eFigure 14. Adjusted HRs for major coronary events (MCE) for (a) short and (b) long vs normal sleep duration by subgroups****


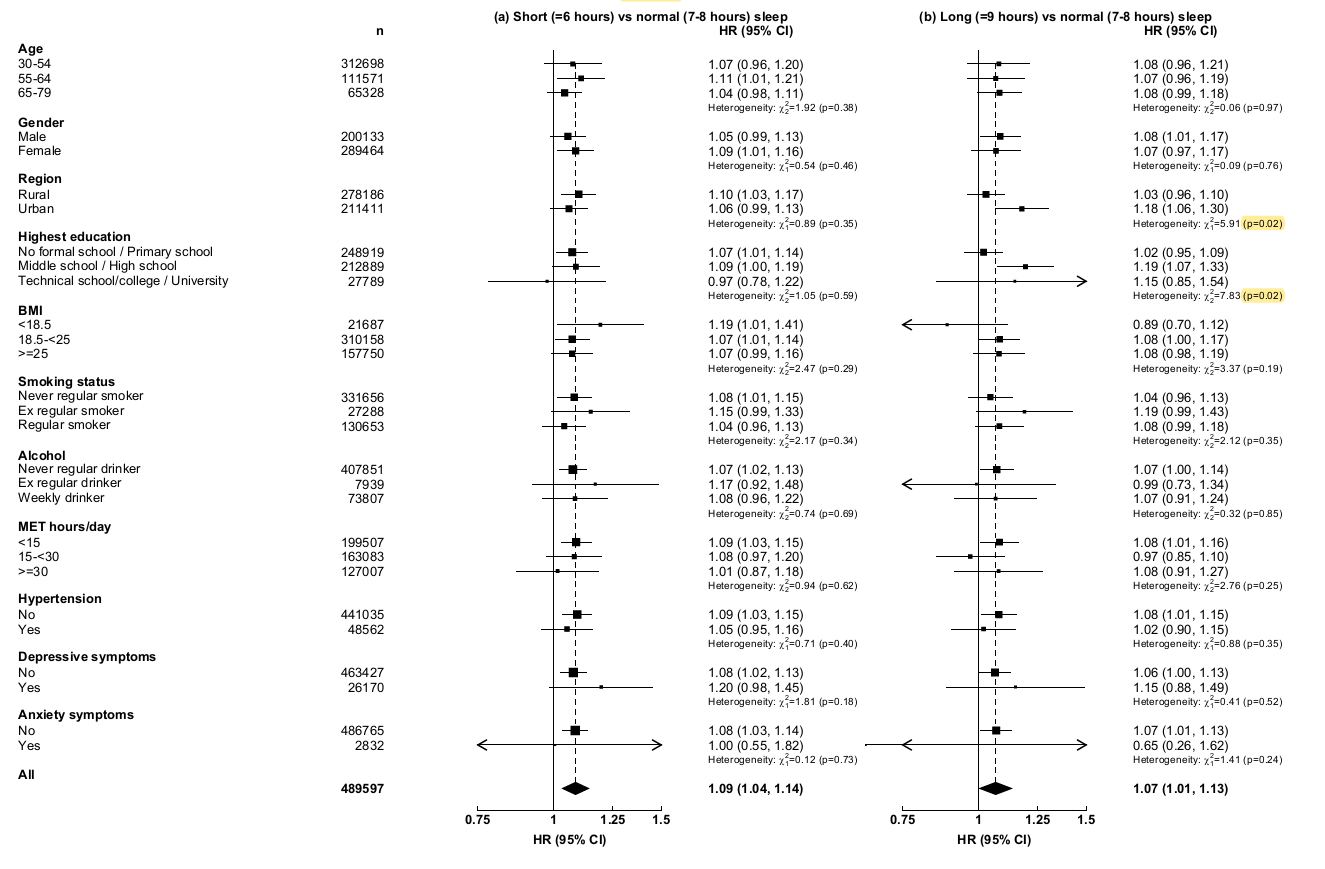


# ****eFigure 15. Adjusted HRs for vascular mortality for (a) short and (b) long sleep duration**** vs normal sleep duration, by subgroups


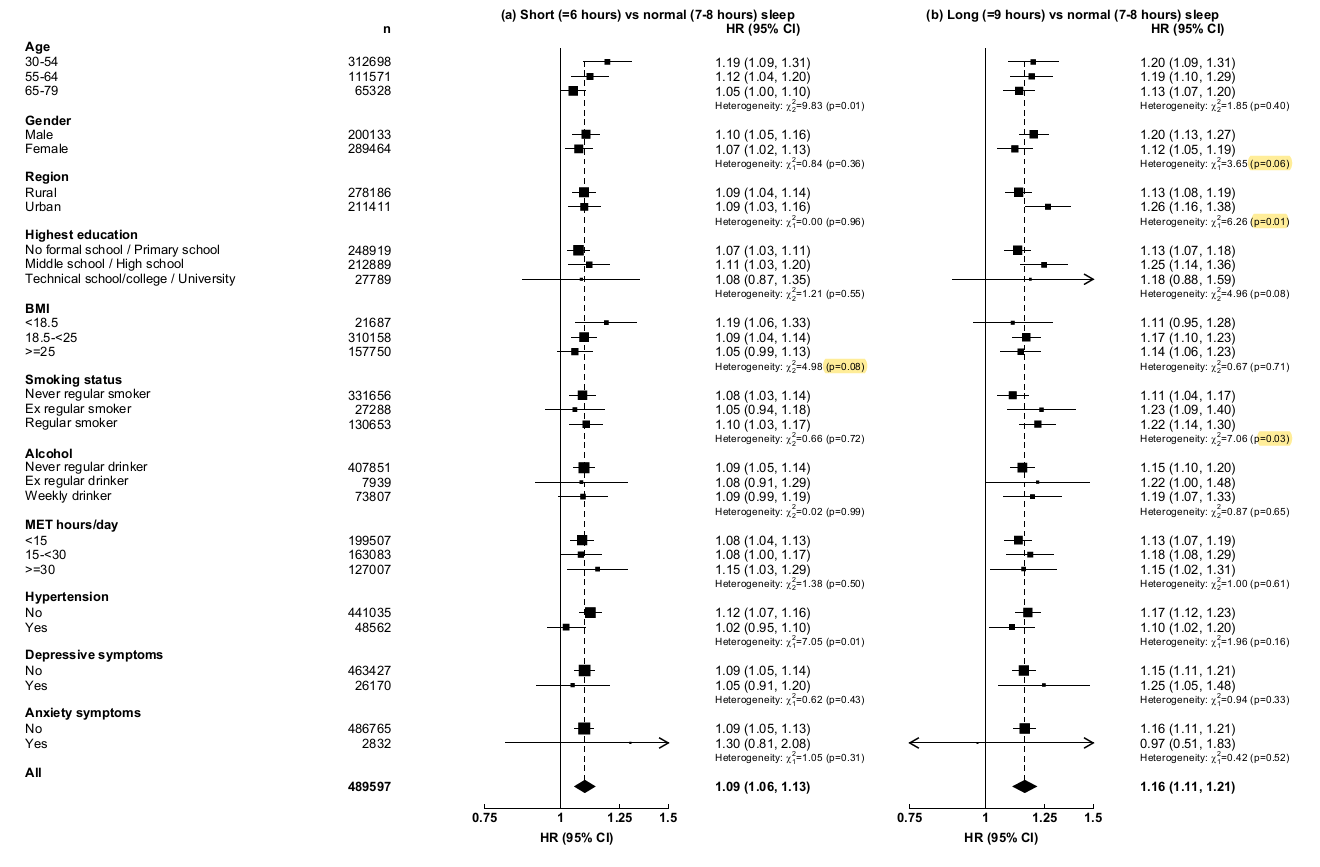

Supplement: Supplementary [file EMS187939-supplement-Supplementary.docx]
